# Insulin-Like Growth Factor-1 Receptor Is Regulated by microRNA-133 during Skeletal Myogenesis

**DOI:** 10.1371/journal.pone.0029173

**Published:** 2011-12-15

**Authors:** Mian-Bo Huang, Hui Xu, Shu-Juan Xie, Hui Zhou, Liang-Hu Qu

**Affiliations:** Key Laboratory of Gene Engineering of the Ministry of Education, State Key Laboratory for Biocontrol, Sun Yat-sen University, Guangzhou, People's Republic of China; Cardiovascular Research Institute Maastricht, Maastricht University, Netherlands

## Abstract

**Background:**

The insulin-like growth factor (IGF) signaling pathway has long been established as playing critical roles in skeletal muscle development. However, the underlying regulatory mechanism is poorly understood. Recently, a large family of small RNAs, named microRNAs (miRNAs), has been identified as key regulators for many developmental processes. Because miRNAs participate in the regulation of various signaling pathways, we hypothesized that miRNAs may be involved in the regulation of IGF signaling in skeletal myogenesis.

**Methodology/Principal Findings:**

In the present study, we determined that the cell-surface receptor IGF-1R is directly regulated by a muscle-specific miRNA, microRNA-133 (miR-133). A conserved and functional binding site for miR-133 was identified in the 3′untranslated region (3′UTR) of IGF-1R. During differentiation of C2C12 myoblasts, IGF-1R protein, but not messenger RNA (mRNA) expression, was gradually reduced, concurrent with the upregulation of miR-133. Overexpression of miR-133 in C2C12 cells significantly suppressed IGF-1R expression at the posttranscriptional level. We also demonstrated that both overexpression of miR-133 and knockdown of IGF-1R downregulated the phosphorylation of Akt, the central mediator of the PI3K/Akt signaling pathway. Furthermore, upregulation of miR-133 during C2C12 differentiation was significantly accelerated by the addition of IGF-1. Mechanistically, we found that the expression of myogenin, a myogenic transcription factor reported to transactivate miR-133, was increased by IGF-1 stimulation.

**Conclusion/Significance:**

Our results elucidate a negative feedback circuit in which IGF-1-stimulated miR-133 in turn represses IGF-1R expression to modulate the IGF-1R signaling pathway during skeletal myogenesis. These findings also suggest that miR-133 may be a potential therapeutic target in muscle diseases.

## Introduction

Skeletal muscle development (myogenesis) is orchestrated by myoblast proliferation, withdrawal from the cell cycle, differentiation and subsequent fusion into multinuclear myotubes. The process of myogenesis requires cooperative actions of the basic helix-loop-helix transcription factors of the MyoD family (MyoD, Myf5, myogenin, MRF4) and other transcription factors, such as members of the MEF2 family (MEF2A-D) [1], which are modulated by various extracellular stimuli and regulated by distinct signaling pathways [2], [3], [4], [5].

The insulin-like growth factor (IGF) signaling pathway is unique because it promotes virtually every biological process, including proliferation, differentiation, growth and survival during embryonic and postnatal muscle development [6]. The actions of the IGFs (IGF-1 and IGF-2) in stimulating intracellular signaling cascades are mediated by the IGF-1 receptor (IGF-1R), a receptor tyrosine kinase. Upon ligand binding, IGF-1R becomes autophosphorylated and induces the phosphatidylinositol 3-kinase (PI3K)/Akt pathway, which is integral to the processes of skeletal muscle development and growth [7], [8]. Disrupted IGF-1R signaling may lead to abnormal muscle development, as shown by the fact that mice carrying a null mutation of the *Igf-1r* gene develop muscle hypoplasia and those lacking IGF-1R in muscle exhibit impaired skeletal muscle development [9], [10]. By contrast, ectopic expression of IGF-1R in muscle results in muscle hypertrophy [11], [12]. Therefore, tight control of the IGF-1R signaling pathway is important for normal muscle cell development. However, the regulatory mechanisms of IGF-1R signaling during muscle development remain unclear.

MicroRNAs (miRNAs) represent a class of ∼22-nucleotide endogenous non-coding RNAs. These molecules typically repress gene expression by base pairing to the 3′untranslated regions (3′UTR) of target messenger RNAs (mRNA), leading to translational repression and/or mRNA degradation in animals [13]. Since their discovery, a cohort of miRNAs have been found to participate in the regulation of various cellular processes, including cell proliferation, differentiation and apoptosis [14], [15]. In particular, spatial- and temporal-specific miRNAs serve as pivotal regulators of tissue determination, differentiation and maintenance [16], [17]. Recently, compelling evidence suggests that signal transduction pathways are prime candidates for miRNA-mediated regulation during embryogenesis or tissue development [18]. Therefore, we hypothesized that miRNAs may be involved in the regulation of IGF-1R signaling during skeletal myogenesis.

In the present study, we found that muscle-specific miR-133 posttranscriptionally represses IGF-1R expression during myogenic differentiation of C2C12 myoblasts by directly binding to its 3′UTR and thus negatively modulating the PI3K/Akt signaling pathway. Furthermore, IGF-1 accelerated induction of miR-133 in differentiating myoblasts, probably through an increase of myogenin protein. Our results reveal a negative feedback mechanism in which IGF-1-stimulated miR-133 is involved in the downregulation of the IGF-1R signaling pathway during skeletal muscle development.

## Results

### IGF-1R is a direct target of miR-133

**Figure 1:**
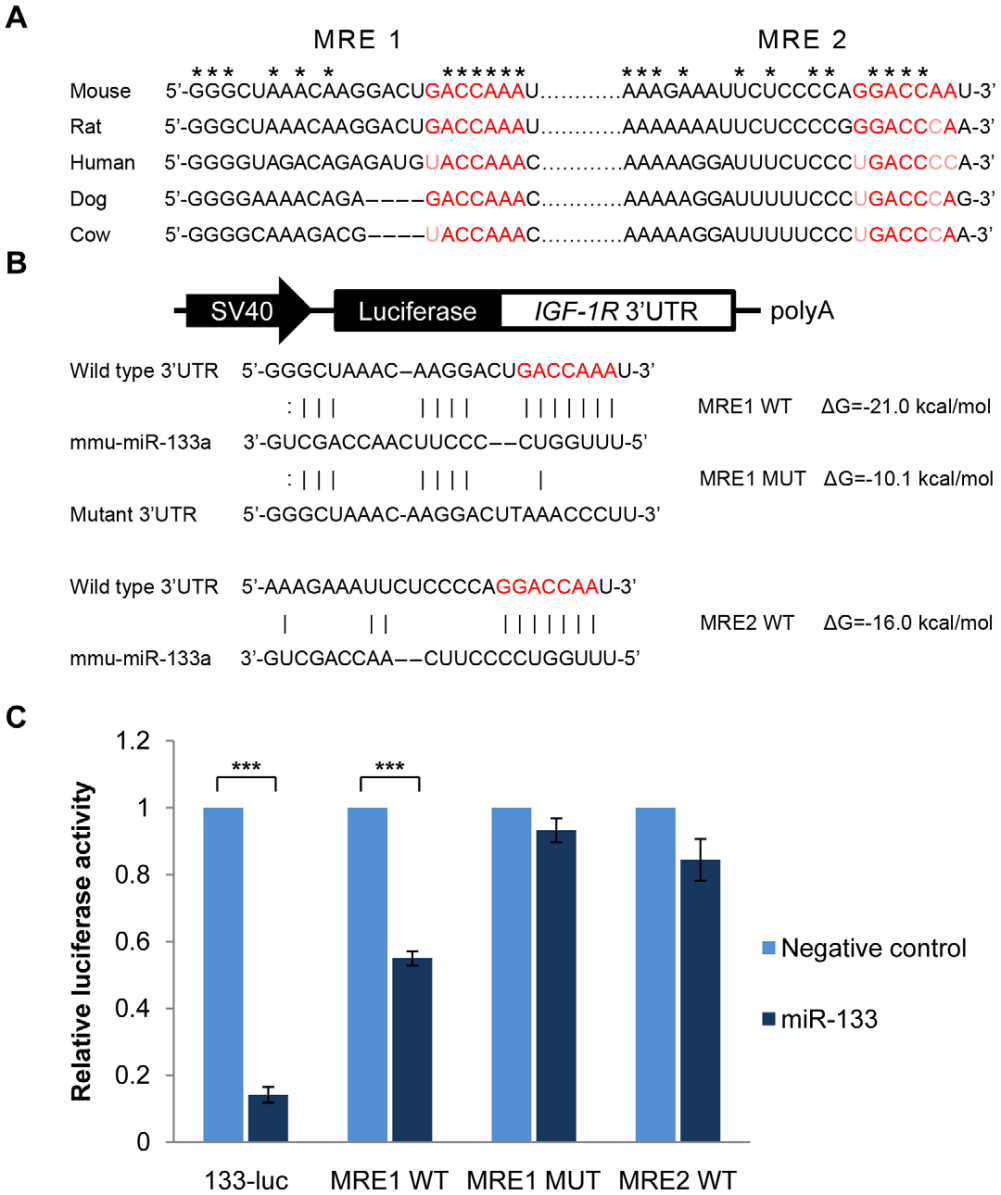
Identification of a functional miR-133 binding site in the IGF-1R 3′UTR. (A) Seed-matched sequences in the IGF-1R 3′UTRs are in red and conserved regions between aligned sequences are indicated by stars. (B) Schematic representation of luciferase reporter constructs. miRNA-mRNA hybridization structures and folding energies were predicted by RNAhybrid. (C) HEK 293T cells were transfected with psiCHECK2 luciferase reporter vectors containing wild type or mutated miR-133 binding sites downstream of the *Renilla* luciferase gene (50 ng), and the internal *Firefly* luciferase gene was used to normalize for transfection efficiency. A pcDNA6.2-miR-133 expression vector or pcDNA6.2-negative control vector was cotransfected (150 ng). Dual-luciferase assays were performed 48 hours after transfection. Normalized luciferase activities of miR-133 transfectants were shown as the percentage relative to pcDNA6.2 transfectant, which was set at 1. Data represent the mean ± standard deviation (S.D.) of three independent experiments. ***p<0.001 vs. pcDNA6.2 transfectants.

To investigate which miRNAs participate in IGF-1R regulation, we screened the 3′UTR of IGF-1R mRNA for potential miRNA binding sites by TargetScan 5.1. Among the miRNAs predicted to target IGF-1R mRNA, we focused on miRNA-133, which is expressed abundantly during muscle development [19]. The mouse IGF-1R transcript was predicted to contain two canonical miR-133 response elements (MREs) in the 3′UTR, suggesting that IGF-1R may be a regulatory target of miR-133. The 3′UTR sequence of mouse IGF-1R was aligned to those of rat, human, dog and cow. The seed-matched region of the miRNA-mRNA interaction is most conserved in MRE 1 (Figure 1A). Predicted hybridization structure also suggested a more favorable folding energy between MRE 1 of mouse IGF-1R mRNA and miR-133 (Figure 1B).

To further investigate whether miR-133 represses IGF-1R directly, fragments of the IGF-1R 3′UTR containing the potential binding sites were inserted downstream of the *Renilla* luciferase gene in the psiCHECK2 reporter vector. As a positive control, activity of the luciferase reporter with the antisense sequence of miR-133 (miR-133-luc) was abrogated by miR-133. Cotransfection of the reporter construct containing wild-type MRE 1 along with the miR-133 expression vector caused a significant reduction of luciferase activity. In contrast, introduction of a mutation in the seed-matched region of MRE 1 abolished the repression by miR-133. However, miR-133 had no effect on the reporter construct containing wild-type MRE 2 (Figure 1C).

### Reciprocal expression of IGF-1R and miR-133 during muscle development and C2C12 cell differentiation

**Figure 2:**
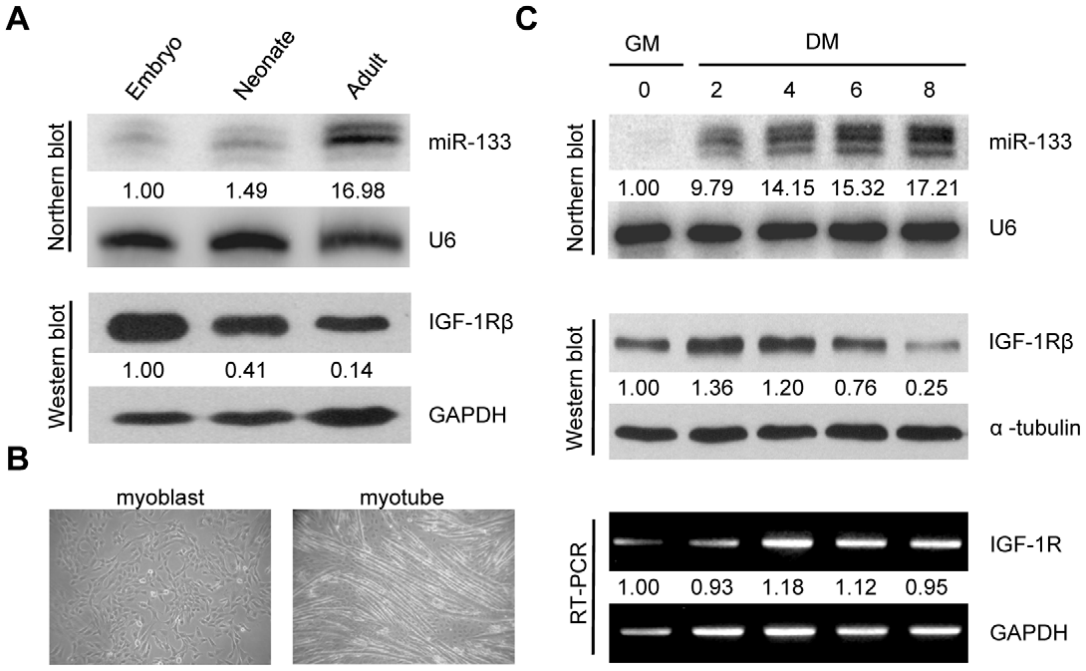
Reciprocal expression of IGF-1R and miR-133 during muscle development and C2C12 cell differentiation. (A) IGF-1R protein and miR-133 levels were determined in hind limb muscles at 18.5 dpc embryos, 2 days postnatal neonates or adults in C57BL/6J mice by western blot or northern blot analysis. GAPDH or U6 snRNA served as loading controls for protein or small RNA. (B) Morphology of proliferating myoblasts maintained in growth medium or differentiated myotubes after serum derivation for 6 days. (C) C2C12 myoblasts were induced to differentiate for up to 8 days. Protein levels were determined by western blotting, miRNA by northern blotting and mRNA by semi-quantitative RT-PCR. Representative results from independent experiments (n≥2) are shown. The numbers below the blots represent relative expression levels. GM represents growth medium and DM represents differentiation medium.

To study the possible regulation of IGF-1R by miR-133, we monitored the expression of IGF-1R protein and miR-133 during muscle development. Muscle tissues were separated from the hind limbs of embryonic, neonatal and adult mice for protein or RNA extraction. Western blotting showed that IGF-1R protein was abundantly expressed in skeletal muscle of 18.5 days post-coitum (dpc) embryos, whereas reduced expression of IGF-1R protein in adult skeletal muscle was observed. In contrast, results from northern blotting showed that miR-133 levels increased dramatically in skeletal muscles as mice grew into adulthood (Figure 2A), displaying an inverse relationship to IGF-1R expression.

We next used C2C12 myoblasts, an established mouse cell line model, to recapitulate the process of myogenic differentiation [20]. C2C12 cells continue to proliferate in high serum conditions but exit from the cell cycle and differentiate into myotubes after serum withdrawal (Figure 2B). During 8 days of culturing in low serum conditions, IGF-1R protein levels increased early and then sharply declined when C2C12 cells differentiated into mature myotubes (Figure 2C). In contrast, IGF-1R transcript remained at a constant level from day 4, suggesting a posttranscriptional regulation of IGF-1R mRNA. Meanwhile, expression of miR-133 simultaneously increased during the differentiation process (Figure 2C).

### MiR-133 negatively modulates IGF-1R/PI3K/Akt signaling through repression of IGF-1R in C2C12 cells

**Figure 3:**
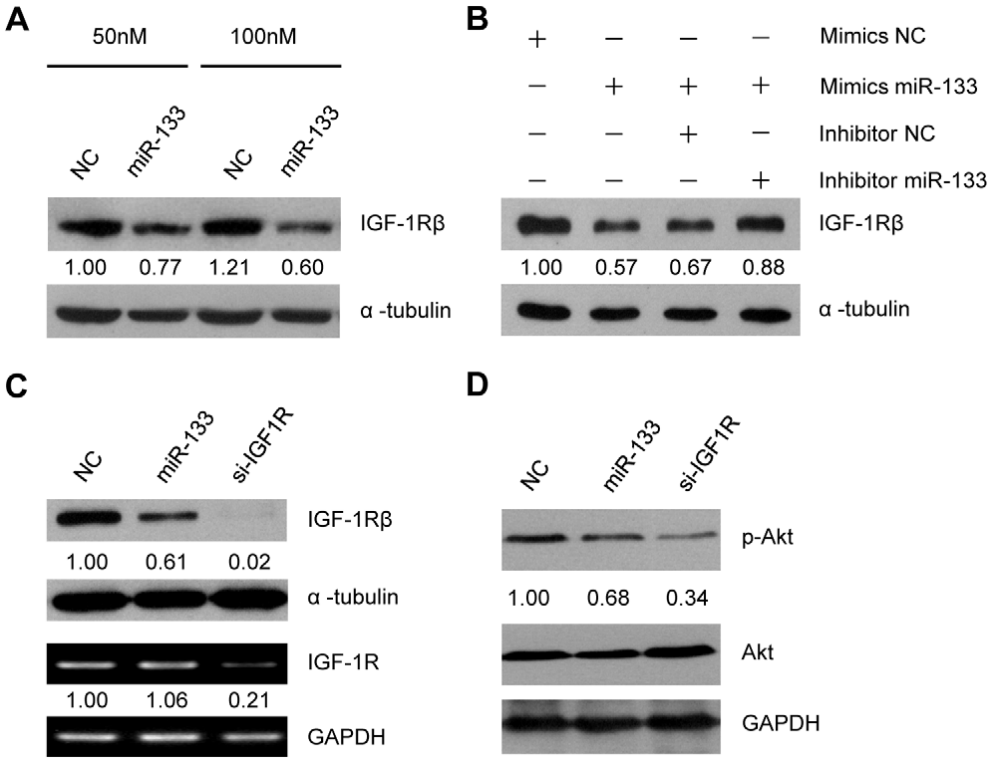
miR-133 negatively regulates the IGF-1R signaling pathway in C2C12 cells. (A) C2C12 cells were transfected with negative control or miR-133 mimics at 50 nM or 100 nM for 36 hours. (B) C2C12 cells were transfected with 50 nM RNA mimics and inhibitors as indicated for 36 hours. (C) C2C12 cells were transfected with 50 nM negative control, miR-133 mimics or anti-IGF-1R siRNA. Proteins were extracted for western blotting against IGF-1R. α-tubulin was served as loading control. The mRNA levels of IGF-1R were determined by semi-quantitative RT-PCR, and GAPDH was used as internal control. (D) C2C12 cells were transfected as in (C). 24 hours after transfection, cells were serum starved for 24 hours and incubated in differentiation medium supplemented with 5 nM IGF-1 for 30 minutes. Proteins were extracted for western blotting against Ser-473 phosphorylated Akt and total Akt. GAPDH was used as a loading control. Representative results from independent experiments (n≥2) are shown. The numbers below the blots represent relative expression levels.

To test whether miR-133 mediates the posttranscriptional regulation of endogenous IGF-1R in muscle, we introduced miR-133 mimics into C2C12 cells. An RNA duplex of scrambled sequence was transfected as negative control. Western blot analysis showed that ectopic expression of miR-133 decreased endogenous IGF-1R protein in a dose-dependent manner in C2C12 cells (Figure 3A). When 2′-O-methylated antisense RNA inhibitors and miR-133 mimics were cotransfected into C2C12 cells, the miR-133 inhibitors, but not the negative control, reversed the repression of IGF-1R expression, further confirming the specific regulation of IGF-1R by miR-133 (Figure 3B). Notably, exogenous miR-133 exerted minor effects on IGF-1R mRNA levels, while small interfering RNA (siRNA) targeting IGF-1R led to dramatic reduction of both IGF-1R mRNA and protein levels in C2C12 cells. These data demonstrate that miR-133 represses IGF-1R at the posttranscriptional level (Figure 3C).

Because the IGF-1-activated IGF-1R/PI3K/Akt signaling pathway is essential for skeletal muscle development, we asked whether miR-133-mediated regulation of IGF-1R protein levels was sufficient to modulate this signaling pathway. Overexpression of miR-133 in C2C12 cells reduced IGF-1-stimulated phosphorylation of Akt at Serine-473, the Akt activation site. Moreover, siRNA targeting of IGF-1R also repressed phosphorylation of Akt (Figure 3D). These results indicate that ectopically expressed miR-133 represses IGF-1R translation to reduce overall PI3K/Akt signaling.

### IGF-1 stimulation potentiates expression of miR-133 during myogenesis

**Figure 4:**
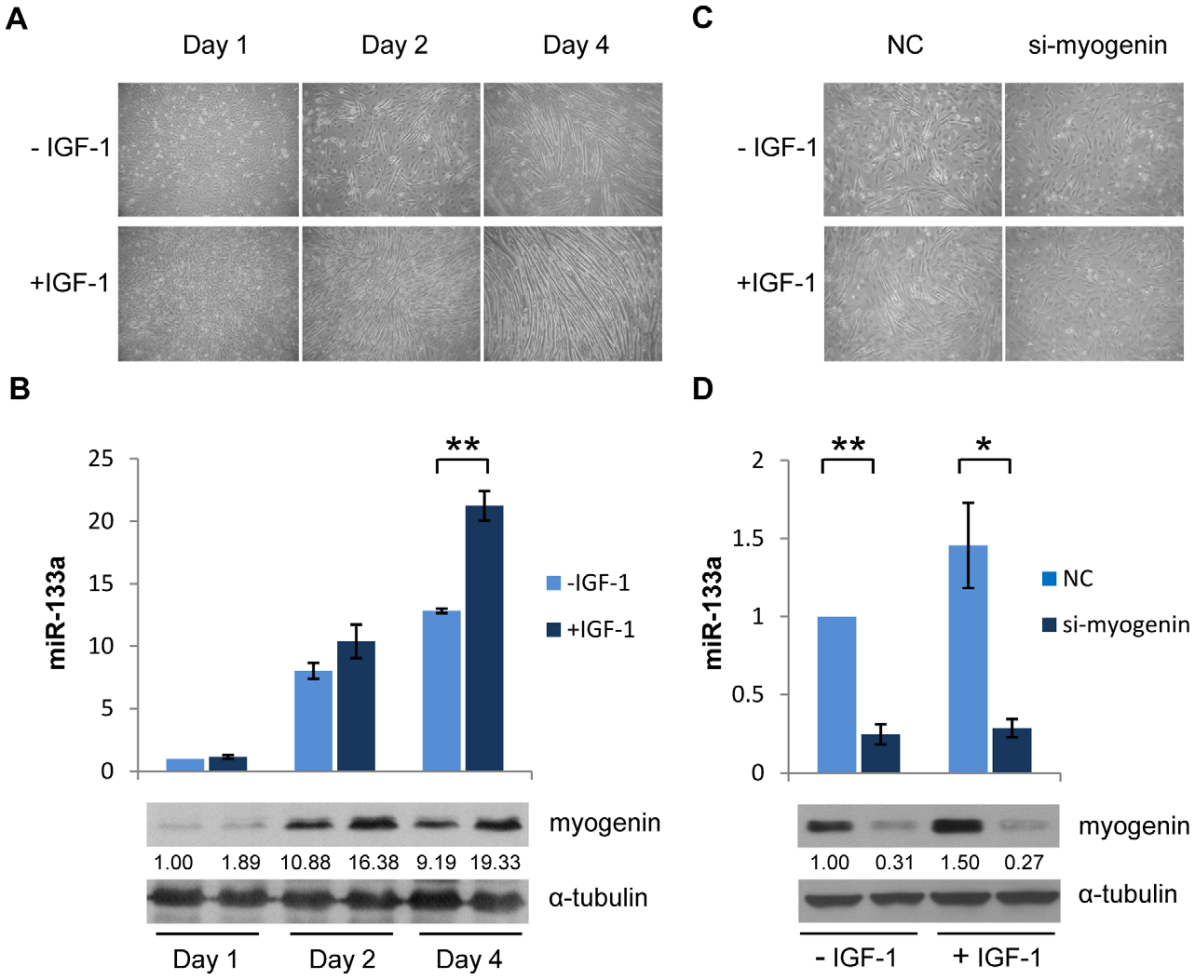
IGF-1 stimulation induces expression of miR-133. (A) C2C12 cells were induced to differentiate in the presence or absence of 5 nM IGF-1. Morphological change was examined by phase contrast microscopy. (B) miR-133 levels normalized to U6 were determined by quantitative RT-PCR. miR-133 levels in day 1 after differentiation induction were set at 1, and relative expression was shown as fold induction. Data represent the mean ± S.D. of three independent experiments. **p<0.01 vs. day 1 without IGF-1 treatment. Protein extracted from differentiating C2C12 cells in the presence or absence of IGF-1 was used for western blot analysis using a myogenin antibody; α-tubulin served as loading control. (C) C2C12 cells were transfected with myogenin siRNA for 24 hours before induced to differentiate in the presence or absence of 5 nM IGF-1 for another 48 hours. Morphological change was shown. (D) Relative miR-133 levels were determined by quantitative RT-PCR. miR-133 levels in negative control transfectants without IGF-1 treatment were set at 1, and relative expression was shown. Data represent mean ± S.D. of three independent experiments. *p<0.05, **p<0.01 vs. negative control transfectants without IGF-1 treatment. Western blot analysis determined the expression of myogenin protein. Representative results from independent experiments (n≥2) are shown. The numbers below the blots represent relative expression levels.

It was observed that plasma levels of IGF-1 increase during development [21], and IGF-1 was found to induce expression of myogenin, a myogenic transcription factor demonstrated to activate miR-133 expression [8], [22]. We thus hypothesized that IGF-1 contributes to miR-133 induction via myogenin during myogenesis. To recapitulate IGF-1-stimulated muscle development, we applied IGF-1 to differentiating C2C12 cells. IGF-1 potently induced myotube formation as shown by the morphology of differentiating C2C12 cells examined by microscopy. Higher cell density and more differentiated myotubes were observed in C2C12 cells treated with IGF-1, demonstrating the positive effects of IGF-1 on muscle cell proliferation and differentiation (Figure 4A). Interestingly, expression of miR-133 displayed faster kinetics in C2C12 cells treated with IGF-1 than those without stimulation, indicating that IGF-1 exerts positive effects on miR-133 expression during myogenesis. Consistently, differentiating C2C12 cells expressed higher levels of myogenin protein in the presence of IGF-1 (Figure 4B). To test whether IGF-1 stimulates miR-133 expression directly through myogenin, we used RNA interference (RNAi) technology to knock down myogenin. An siRNA targeting myogenin significantly hindered the differentiation of C2C12 cells even in the presence of IGF-1 (Figure 4C). As a result of the repression of myogenin by siRNA, miR-133 expression decreased in differentiating C2C12 cells. IGF-1 treatment did not reverse such repressive effect (Figure 4D), indicating that myogenin is necessary for IGF-1-induced miR-133 expression.

## Discussion

**Figure 5:**
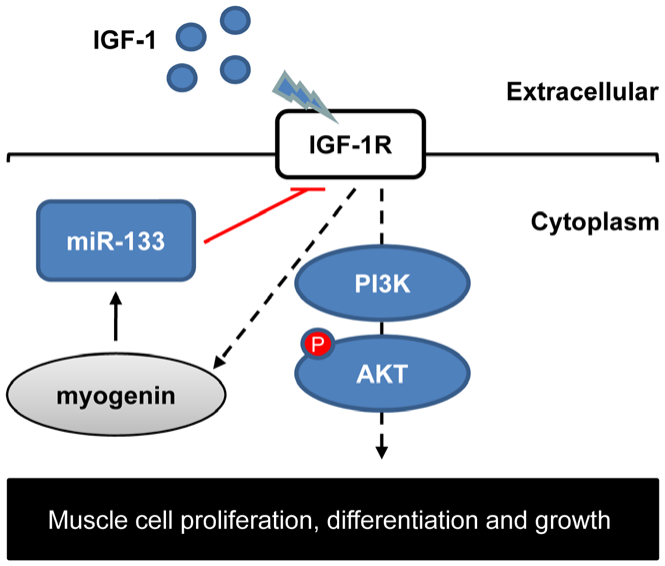
Model of the myogenin/miR-133/IGF-1R negative regulatory circuit in muscle. miR-133 represses IGF-1R at the posttranscriptional level and negatively regulates the IGF-1R/PI3K/Akt signaling pathway, which is involved in skeletal muscle proliferation, differentiation and hypertrophy. Extracellular IGF-1 accelerates miR-133 expression via myogenin induction.

This study identified a novel function of miR-133 during skeletal myogenesis of negative regulation of IGF-1R and the PI3K/Akt signaling pathway. In addition, we showed that IGF-1 stimulates the upregulation of miR-133 during skeletal myogenic differentiation. Our findings support a negative feedback circuit in the regulation of IGF-1R signal transduction during skeletal myogenesis, as illustrated in Figure 5.

The expression level of IGF-1R is critical for the regulation of muscle development because it directly influences the intracellular responsiveness of muscle cells to the extracellular IGF signal. Both transcriptional and posttranscriptional regulatory mechanisms are essential for tight control of the expression of IGF-1R [23], [24]. Here we show that the gradual reduction of IGF-1R protein levels during C2C12 differentiation is regulated by miR-133, which is significantly induced during the same process. Through inhibition of IGF-1R protein expression, miR-133 downregulates the phosphorylation of Akt, which plays a key role in multiple cellular processes such as glucose metabolism, cell proliferation, differentiation, growth and apoptosis [25], [26]. Therefore, miR-133 may regulate various intracellular responses controlled by the IGF-1R/PI3K/Akt signaling pathway during myogenesis. Supportive of such role is the evidence that phosphorylation of Akt (at Ser-473) is shown to be gradually silenced during C2C12 differentiation [27]. During our study, others have found that another muscle-specific miRNA, miR-1, also directly targets IGF-1R [27]. The two most abundant miRNAs in muscle are employed to posttranscriptionally repress IGF-1R expression during skeletal muscle development, implying that the IGF-1R expression level may be a decisive factor in myogenesis.

As one of the most abundant myogenic microRNAs, miR-133 has been reported to regulate multiple targets which are involved in skeletal muscle development. The first one identified is the transcription factor serum response factor (SRF), which is critical for muscle proliferation and differentiation [19]. Other target genes like alternative splicing factor neuron-polypyrimidine tract-binding protein (nPTB) and energy expenditure regulator uncoupling protein 2 (UCP2) were also verified [28], [29]. These findings emphasized the important regulatory role of miR-133 in modulating muscle development.

Several myogenic transcription factors have been shown to transactivate miR-133 in muscle cells [22], [30], [31]. However, the upstream signaling pathway has not been determined. Interestingly, we found that stimulation of differentiating C2C12 cells with IGF-1 increased the expression of both miR-133 and the transcription factor myogenin (Figure 4B), which has been shown to directly regulate miR-133 transcription [8], [22]. This finding reveals that miR-133 forms a feedback circuit with the IGF-1R signaling pathway in muscle cells (Figure 5). This notion is supported by the observation that IGF-1R and miR-133 were reciprocally expressed (Figure 2A) when circulating IGF-1 levels increased as the mice matured [21]. Such a homeostatic regulatory mechanism may play an important role in normal muscle development. Coincidentally, similar regulatory circuits has been reported recently in a neuron-like differentiation model, suggesting that miRNA-mediated negative feedback loops may be general mechanisms for the regulation of IGF-1R signaling in various tissues [32].

miRNAs exert profound effects by cooperatively regulating multiple components in the same signaling pathway. Apart from the IGFs in the microenvironment, the endogenously expressed IGFs also play important roles in myogenesis [33]. Therefore, muscle cells may also utilize miRNAs to tightly control the expression of IGF genes. Recently, miR-1 and miR-125b have been reported to negatively regulate IGF-1 and IGF-2 respectively in myogenesis [27], [34]. Thus, while miR-1 and miR-125b reduce intracellular production of IGFs, cotranscribed miR-133 and miR-1 repress IGF-1R and modulate muscle cell responsiveness to circulating (endocrine) or local (autocrine/paracrine) IGF stimulation. By contrast, muscle-enriched miR-486 facilitates the IGF-1-activated IGF-1R/PI3K/Akt signaling by directly targeting to the negative signaling regulators PTEN and Foxo1a [35]. Therefore, the miRNA-mediated posttranscriptional regulatory network could be of significant importance in balancing the activities of IGFs in muscle cells (Figure S1).

Deregulated IGF-1R signaling can result in muscle pathogenesis. Rhabdomyosarcoma (RMS) is a pediatric soft-tissue sarcoma that arises from dysregulated proliferation and differentiation of skeletal muscle progenitors. IGF-1R expression is elevated in RMS tissues and is considered a key initiator of oncogenic transformation of muscle cells [24]. Recently, miR-1 and miR-133 were found to be dramatically reduced in RMS cell lines, suggesting a pathological link between these miRNAs and RMS [36]. Therefore, our data suggest that reduced miR-133 expression may be responsible for IGF-1R elevation in RMS, thus leading to increased proliferation and blocked differentiation. Compromised IGF-1R signaling has also been implicated in muscle hypertrophy, atrophy and age-associated sarcopenia [37], [38]. A work-induced muscle hypertrophy model has demonstrated that miR-133 and miR-1 expression is reduced in rodent skeletal muscle [39]. Although direct evidence remains to be provided, our findings and those of other studies have suggested that the deregulation of miR-133 and miR-1 may be critical for the pathological mechanism of these muscle diseases. This also raises the possibility that muscle-specific miRNAs may serve as potential therapeutic targets for muscular disorders. Further studies are needed to fully elucidate miRNA-involved physiological or pathological regulatory mechanisms in skeletal muscle.

## Materials and Methods

### Ethic statement

This study was carried out in strict accordance with the recommendations in the Guide for the Care and Use of Laboratory Animals by the National Academy of Sciences. The protocol was approved by the Committee on the Ethics of Animal Experiments of Sun Yat-sen University (Permit Number: 2009-0011). All efforts were made to minimize suffering.

### Cell culture and transfection

C2C12 myoblasts obtained from the Cell Bank of Chinese Academy of Sciences (Shanghai, China) were maintained at subconfluent densities in growth medium (GM) supplemented with 10% fetal bovine serum (Gibco, Carlsbad, CA) in Dulbecco's modified Eagle's medium (DMEM). Near-confluent cells were induced to differentiate in differentiation medium (DM) consisting of DMEM plus 2% horse serum (Gibco, Carlsbad, CA) for 8 days. LONG™ R^3^ IGF-1 (Sigma-Aldrich, St. Louis, MO) was added to differentiation medium to a final concentration of 5 nM. HEK 293T cells obtained from the Cell Bank of Chinese Academy of Sciences (Shanghai, China) were maintained in DMEM supplemented with 10% fetal bovine serum. RNA oligonucleotides or DNA vectors were transfected with Lipofectamine™ 2000 (Invitrogen, Carlsbad, CA) following the manufacturer's instructions. RNA duplexes were synthesized by GenePharma (Shanghai, China), and 2′-O-methylated miRNA inhibitors were synthesized by RiboBio (Guangzhou, China). RNA oligonucleotides are listed in table S1.

### Plasmid constructs

To generate a miR-133 expression vector, a 300-bp fragment containing the miR-133-1 genomic sequence was amplified by PCR from C57BL/6J mouse genomic DNA and cloned into a modified pcDNA6.2-GW/EmGFP vector (Invitrogen, Carlsbad, CA) between EcoRI and XhoI sites [17]. Fragments of 3′UTRs containing putative miR-133 binding sites were amplified by PCR and cloned into the psiCHECK2 vector (Promega, Madison, WI) between SalI/XhoI and NotI sites downstream of the *Renilla* luciferase gene. Point mutations were introduced by PCR using primers with the mutated sequence at the 5′ end. All constructs were verified by DNA sequencing. Primers are listed in table S1.

### Dual-luciferase reporter assays

HEK 293T cells were allowed to attach overnight in 48-well plates (4×10^4^ cells in each well). On the following day, the cells were cotransfected with either 150 ng pcDNA6.2-miR-133a or pcDNA6.2-negative control expression vectors and 50 ng wild-type or mutant psiCHECK2-3′UTR reporter vectors. The cells were harvested, and dual-luciferase assays were performed 48 hours after transfection using the Dual-Luciferase Reporter Assay System (Promega, Madison, WI). *Renilla* luciferase activity was normalized to *Firefly* luciferase expression for each sample to account for differences in transfection efficiency.

### RT-PCR and northern blot analysis

Total RNA was extracted from mouse tissues or C2C12 cells with Trizol reagent (Invitrogen, Carlsbad, CA). For RT-PCR, total RNA was reverse transcribed using the PrimeScript reverse transcription reagent kit (Takara Biotech. Co., Ltd., Dalian, China) and amplified by PCR using specific primers. For quantitative RT-PCR of miRNA, RNA was reverse transcribed using a stem-loop primer as described elsewhere [40] and PCR amplified with the SYBR™ Premix ExTaq kit (Takara Biotech. Co., Ltd., Dalian, China). Fold changes in gene expression were calculated as 2^−ΔΔCt^. For northern blot analysis, RNA was separated on 15% polyacrylamide denaturing gels and transferred to Hybond-N nylon membranes (Amersham Biosciences, Buckinghamshire, England). Membranes were hybridized using specific probes with [γ-^32^P] ATP labeling at the 5′end. Membranes were exposed with a phosphorimager plate and visualized by a Typhoon 8600 imager (Amersham Biosciences, Buckinghamshire, England). All primers are listed in table S1.

### Western blot analysis

Skeletal muscle was dissected from hind limbs of C57BL/6J mice and ground to powder in liquid nitrogen. Proteins from mouse tissues or C2C12 cells were extracted in RIPA buffer (50 mM Tris-HCl, pH 8.0, 150 mM Sodium chloride, 1% NP-40, 0.5% sodium deoxycholate, 0.1% SDS, 2 mM EDTA and 1 mM PMSF), and protein concentration was determined by the BCA protein assay kit (Pierce Biotechnology Inc., Rockford, IL). Proteins were separated on 10% SDS-PAGE gels and transferred to Hybond-P PVDF membranes (Amersham Biosciences, Buckinghamshire, England). The membranes were incubated with primary antibodies to IGF-1Rβ, α-tubulin, p-Akt (Ser-473), Akt (pan), GAPDH from Cell Signaling Technology (Beverly, MA), or myogenin from Santa Cruz Biotechnology Inc. (Santa Cruz, CA). The blots were then incubated with horseradish peroxidase-conjugated secondary antibodies and visualized using commercial ECL kits (Cell Signaling Technology, Beverly, MA).

### Bioinformatic and statistical analysis

Prediction of miRNA target sites in 3′UTRs was performed by TargetScan 5.1 Mouse (http://www.targetscan.org) [41]. The hybridization structure between miRNA and its putative binding site was analyzed with RNAhyrid (http://bibiserv.techfak.uni-bielefeld.de/rnahybrid/) [42]. Results from northern blot, western blot and RT-PCR were quantified using the densitometric image analysis software Quantity One from Bio-Rad (Richmond, CA). Normalization was made against internal controls. Data are presented as the mean ±standard deviation and subjected to Student's t test; a value of *p*<0.05 was considered statistically significant.

## Supporting Information

Figure S1 **Coordinate regulation of IGF-1R/PI3K/Akt pathway by miRNAs in muscle.** miR-1, miR-133 and miR-125b negatively regulate IGF-1R/PI3K/Akt signal transduction by reducing ligand production or IGF-1R protein levels. miR-486 promotes this pathway by repressing negative regulators.
(TIF)

Table S1 **Sequence of RNA and DNA Oligonucleotides.**
(DOC)
