# Supplementary figures and images for: Insulin-Like Growth Factor-1 Receptor Is Regulated by microRNA-133 during Skeletal Myogenesis
Source: PLoS One. 2011 Dec 15;6(12):e29173. doi: 10.1371/journal.pone.0029173 (PMC3240640; doi:10.1371/journal.pone.0029173)

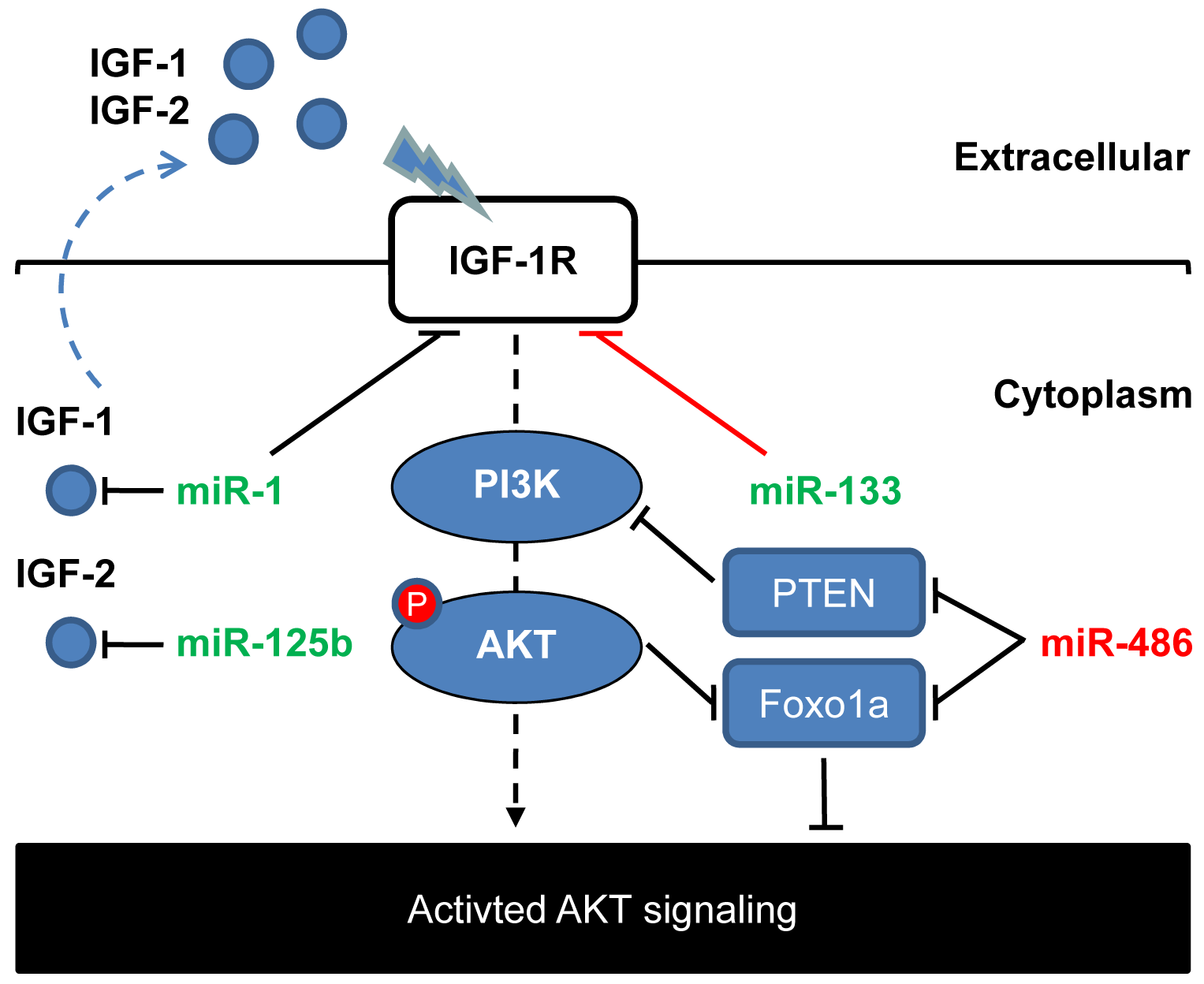

Supplement: Figure S1 — Coordinate regulation of IGF-1R/PI3K/Akt pathway by miRNAs in muscle. miR-1, miR-133 and miR-125b negatively regulate IGF-1R/PI3K/Akt signal transduction by reducing ligand production or IGF-1R protein levels. miR-486 promotes this pathway by repressing negative regulators. (TIF) [file pone.0029173.s001.tif]
