# Supplementary material for: Insulin-Like Growth Factor-1 Receptor Is Regulated by microRNA-133 during Skeletal Myogenesis
Source: PLoS One. 2011 Dec 15;6(12):e29173. doi: 10.1371/journal.pone.0029173 (PMC3240640; doi:10.1371/journal.pone.0029173)
Supplement: Table S1 — Sequence of RNA and DNA Oligonucleotides. (DOC) [file pone.0029173.s002.doc]

**Table S1. Sequence of RNA and DNA Oligonucleotides**

| **Name** | **Forward (5’-3’)** | | **Reverse (5’-3’)** |
| --- | --- | --- | --- |
| **RNA oligonucleotides** | | | |
| Negative control-mimics | UUCUCCGAACGUGUCACGUdTdT | | ACGUGACACGUUCGGAGAAdTdT |
| miR-133a-mimics | UUUGGUCCCCUUCAACCAGCUG | | GCUGGUUGAAGGGGACCAAAUU |
| si-IGF1R | GAACCUUCGUCUCAUCUUAdTdT | | UAAGAUGAGACGAAGGUUCdTdT |
| si-myogenin | GUAAGAGGAAGUCUGUGUCdTdT | | GACACAGACUUCCUCUUACdAdC |
| miR-133a-inhibitor | CAGCUGGUUGAAGGGGACCAAA | |  |
| **Primers for vector construction** | | | |
| pcDNA6.2-miR-133a | CGTGAATTCACACTAGTGTGGGAACCTCT | | TCTGCTCGAGAGGTTGACAGTTGCTAGGTA |
| psiCHECK2-133-luc | TCGAGCAGCTGGTTGAAGGGGACCAAAGTTTAAACCAGCTGGTTGAAGGGGACCAAAGC | | GGCCGCTTTGGTCCCCTTCAACCAGCTGGTTTAAACTTTGGTCCCCTTCAACCAGCTGC |
| psiCHECK2-MRE1-WT | CAGTGTCGACTTGAGAGTCCCAATGTGTGC | | CAGTGCGGCCGCGCAGGTATGTGCGAGAGGAT |
| psiCHECK2-MRE1-MUT | AAGGACTTAAACCCTCTTGAGGCTGGAAGGCCAG | | CTCAAGAGGGTTTAAGTCCTTGTTTAGCCCTGGG |
| psiCHECK2-MRE2-WT | CAGTCTCGAGTGTGTCCACATTTGTGTCTGC | | ATAAGCGGCCGCCACTCCACAGGGAACAGTGA |
| **Primers for mRNA RT-PCR** | | | |
| IGF-1R | TTGCCCTAAAACTGAAGCTGA | | GTTCTCGCAAAGACGAAGTTG |
| GAPDH | ATCACTGCCACCCAGAAGACT | | AGGTGGAAGAGTGGGAGTTGC |
| **Primers for miRNA RT-PCR** | | | |
| miR-133a-RT | GTCGTATCCAGTGCAGGGTCCGAGGTATTCGCACTGGATACGACCAGCTG | | |
| miR-133a | GTCATTTGGTCCCCTTCAAC | | GTGCAGGGTCCGAGGT |
| U6 | CGCTTCGGCAGCACATATAC | | TTCACGAATTTGCGTGTCAT |
| **Primers for northern blot** | |  | |
| miR-133a | CAGCTGGTTGAAGGGGACCAAA | | |
| U6 | GAATTTGCGTGTCATCCTTGCG | | |
